# Barriers to Protocol Adherence in Emergency Departments and Evidence-Based Strategies for Successful Implementation: A Scoping Review

**DOI:** 10.3390/jcm15166420

**Published:** 2026-08-19

**Authors:** Petruta Anca Morosan, Tudor Ovidiu Popa, Paul Nedelea, Amelian Bobu, Andrei Ionut Cucu, Catalin Bouros, Viorica Popa, Anca Haisan, Gabriela Grigorasi, Mihaela Corlade Andrei, Diana Cimpoesu

**Affiliations:** 1 Faculty of Medicine, Grigore T. Popa University of Medicine and Pharmacy Iași, 700115 Iasi, Romania; petruta-anca.morosan@umfiasi.ro (P.A.M.); paul-lucian.nedelea@umfiasi.ro (P.N.); amelian.bobu@gmail.com (A.B.); catalin.bouros@umfiasi.ro (C.B.); viorica.popa@umfiasi.ro (V.P.); anca.haisan@umfiasi.ro (A.H.); gabriela-raluca.grigorasi@umfiasi.ro (G.G.); mihaela.corlade@umfiasi.ro (M.C.A.); carmen.cimpoesu@umfiasi.ro (D.C.); 2 “St. Spiridon” County Emergency Clinical Hospital, 700111 Iasi, Romania; 3 Faculty of Medicine and Biological Sciences, Stefan cel Mare University of Suceava, 720229 Suceava, Romania; andrei.cucu@usm.ro; 4 Emerald Hospital Iasi, 700115 Iasi, Romania; 5 “Dr. Iacob Czihac” Military Emergency Clinical Hospital, 700112 Iasi, Romania

**Keywords:** emergency department, protocol adherence, clinical practice guidelines, implementation science, implementation strategies, cognitive overload, burnout, clinical decision support, patient safety, quality of care

## Abstract

**Background**: Emergency departments (EDs) operate under severe time pressure, diagnostic uncertainty, and resource constraints, making the consistent application of clinical protocols challenging. This scoping review aimed to map the barriers to protocol adherence and the strategies reported to support implementation in emergency care. **Methods**: PubMed/MEDLINE, Scopus, and Web of Science were searched for publications from January 2000 to June 2026. Following predefined eligibility criteria, 58 publications were included and charted according to clinician-related, guideline-related, patient-related, and organizational determinants, and we reported the implementation strategies. No formal design-specific risk-of-bias or certainty-of-evidence assessment was performed; therefore, the synthesis was intended to map the available evidence rather than establish the comparative effectiveness. **Results**: Commonly reported barriers included limited guideline knowledge and clinical experience, cognitive overload and occupational fatigue, poor guideline usability and workflow compatibility, patient communication difficulties and clinical complexity, overcrowding, staffing shortages, and limited organizational support. The reported strategies included education and simulation, audit and feedback, clinical decision support, workflow redesign, multidisciplinary collaboration, and leadership engagement. However, the heterogeneity in study designs, clinical settings, definitions of adherence, and reported outcomes precluded ranking these strategies or determining whether particular combinations were superior. **Conclusions**: Protocol adherence in EDs appears to be shaped by interacting clinician-related, guideline-related, patient-related, and organizational factors. The identified strategies may support implementation, but their relative and comparative effectiveness remains uncertain. Digital health and artificial intelligence should be considered priorities for prospective evaluation rather than established solutions.

## 1. Introduction

Emergency departments (EDs) constitute the cornerstone of acute healthcare systems, providing continuous care for patients with undifferentiated and potentially life-threatening conditions [1]. The demand for emergency care has increased steadily worldwide in recent decades [2]. Across Europe, approximately 25% of the population attends an emergency department each year, accounting for an average of 304 emergency visits per 1000 inhabitants, while ED attendances continue to rise by approximately 2–3% annually [3,4]. Furthermore, 70–80% of all hospital admissions are generated through emergency departments, highlighting their pivotal role in acute care delivery [4]. Similar trends have been observed in the United States, where annual ED visits increased from 128.9 million in 2010 to 144.8 million in 2016, representing a cumulative growth of 12.3% (a compound annual growth rate of 1.95%), substantially exceeding the population growth during the same period [2,5]. More recently, approximately 140 million emergency department visits were recorded in 2021, corresponding to 43 visits per 100 population [2,6,7]. This sustained increase in patient volume, combined with population aging, increasing multimorbidity, workforce shortages, and limited inpatient capacity, has transformed emergency department overcrowding into a major global public health challenge, placing considerable pressure on emergency clinicians and healthcare systems [8,9].

In this increasingly demanding environment, the delivery of high-quality emergency care depends on the consistent application of evidence-based clinical practice guidelines and standardized protocols [10]. These tools provide structured recommendations for the assessment and management of time-critical conditions, supporting rapid clinical decision-making, reducing unwarranted variations in practice, and promoting safe, efficient, and standardized patient care [11,12]. Adherence to evidence-based protocols has consistently been associated with improved quality of care, fewer medical errors, shorter treatment delays, and lower morbidity and mortality across a wide range of emergency conditions [11,13,14].

Despite the well-established benefits of evidence-based protocols, their implementation in emergency departments remains inconsistent [14,15]. Studies evaluating adherence have consistently demonstrated substantial variability across multiple time-critical conditions, reflecting a persistent evidence-to-practice gap in emergency medicine [14]. In a systematic review of prehospital and emergency care, Ebben et al. reported adherence rates ranging from 0% to 98% [16]—Ebben, R.H et al., 2013. More recently, a scoping review of 64 studies confirmed marked variation in the implementation and use of clinical pathways in emergency departments and identified persistent organizational, healthcare professional-related, and pathway-specific barriers [14]. A persistent evidence-to-practice gap is consistently observed across a wide range of emergency conditions. For example, for sepsis, an international study involving 618 hospitals from 62 countries reported complete compliance with the Surviving Sepsis Campaign 3-h and 6-h bundles in only 19% and 36% of patients, respectively, despite significantly lower hospital mortality among patients receiving guideline-compliant care [17]—Rhodes, A. et al., 2015. More recent evidence supports the continued importance of protocolized sepsis care: a 2024 systematic review and meta-analysis including 22 studies and 19,580 emergency department patients found that sepsis alert systems were associated with improved sepsis-bundle adherence and lower mortality [18]. Similarly, adherence to STEMI protocols remains suboptimal, with just over half of EMS-transported patients achieving the recommended door-to-balloon time of ≤90 min [19]. More recent evidence also indicates persistent delays, with a mean door-to-balloon time of 93.6 ± 38.31 min reported among ambulance-transported STEMI patients [20]. Comparable variability has also been reported in trauma systems, where adherence to trauma triage protocols ranges from 21% to 93% across different healthcare settings. The decision of ambulance personnel regarding the referral of trauma patients to appropriate centers is essential for prognosis, and the optimization of these algorithms through support applications has significantly reduced triage errors in recent practice [21,22]. Emergency departments represent one of the most challenging environments for the implementation of evidence-based protocols [12]. Unlike other hospital settings, emergency clinicians must simultaneously evaluate patients with undifferentiated presentations, rapidly recognize life-threatening conditions, prioritize competing clinical demands, and initiate time-critical interventions under considerable diagnostic uncertainty. This process is further complicated by overcrowding, frequent interruptions, cognitive overload, limited resources, staffing shortages, and continuous workflow disruptions, all of which may adversely affect adherence to standardized protocols [8,23,24].

The principal constructs were operationally distinguished in this review for clarity. A clinical practice guideline refers to a systematically developed set of recommendations intended to support clinical decisions in defined circumstances, whereas a protocol refers to a more prescriptive sequence of actions that translates recommendations into locally applicable practice [25]. Grouping clinical interventions into care bundles has proven to be an effective strategy for improving safety indicators and reducing costs associated with prolonged hospitalization. A care bundle comprises a small set of evidence-informed practices intended to be performed collectively and reliably [26,27], while a clinical pathway organizes the sequence and timing of multidisciplinary care for a defined patient group. A clinical decision-support tool provides patient-specific information or recommendations at the point of care. The implementation of clinical decision support systems (CDSS) facilitates compliance with current medical guidelines and standardizes the quality of therapeutic action at the point of care [28,29]. Adherence is used as an umbrella term and is reported according to the definitions employed in the included publications, encompassing institutional adoption, clinician behavior, completion of individual protocol components, and all-or-none bundle compliance [30]. These outcomes were not considered directly interchangeable because they differ in their unit of measurement, denominator, and required threshold.

Despite the availability of well-established evidence-based protocols, translating guideline recommendations into routine emergency department practice remains a persistent challenge. This review aims to map the barriers to protocol adherence and to discuss evidence-based interventions that may facilitate successful implementation in routine clinical practice.

The evidence reviewed shows that adherence to guidelines and protocols is influenced by the complex interaction of clinician-related, guideline-related, patient-related, and organizational factors, and implementation strategies should be tailored to locally identified barriers and embedded within existing clinical processes.

## 2. Materials and Methods

This review was designed as a scoping review to determine the range and characteristics of the evidence on adherence to evidence-based clinical protocols in emergency departments (EDs) and related acute care settings. The review focused on identifying how protocol adherence has been studied, the determinants of non-adherence that have been reported, the implementation strategies used to improve adherence, and the remaining gaps in the literature.

The methodology was informed by established scoping review guidance, using the Preferred Reporting Items for Systematic Reviews and Meta-Analyses extension for Scoping Reviews (PRISMA-ScR). The review questions were as follows: *What evidence exists regarding adherence to evidence-based clinical protocols in emergency departments? What barriers, facilitators, and implementation strategies have been described?*

A structured literature search was conducted in PubMed/MEDLINE, Scopus, and Web of Science. The search included publications from January 2000 to June 2026, with the final search conducted in June 2026. The search concepts were organized around three domains: emergency care setting, protocol or guideline adherence, and implementation barriers or strategies. The year 2000 was selected to capture the period during which evidence-based emergency medicine, standardized clinical protocols, electronic decision support, and implementation science underwent substantial development. Authors A.B. and A.C. were responsible for the database searches and title and abstract screening, and P.M. was in charge of the full-text assessment and settling the differences in opinion.

The search terms included combinations of controlled vocabulary and free-text terms such as emergency department, emergency medicine, clinical protocol, clinical practice guideline, protocol adherence, guideline adherence, implementation, compliance, barriers, facilitators, overcrowding, burnout, workflow, communication, patient safety, and quality of care. Boolean operators, truncation, phrase searching, and database-specific syntax were used where appropriate.

**Figure 1:**
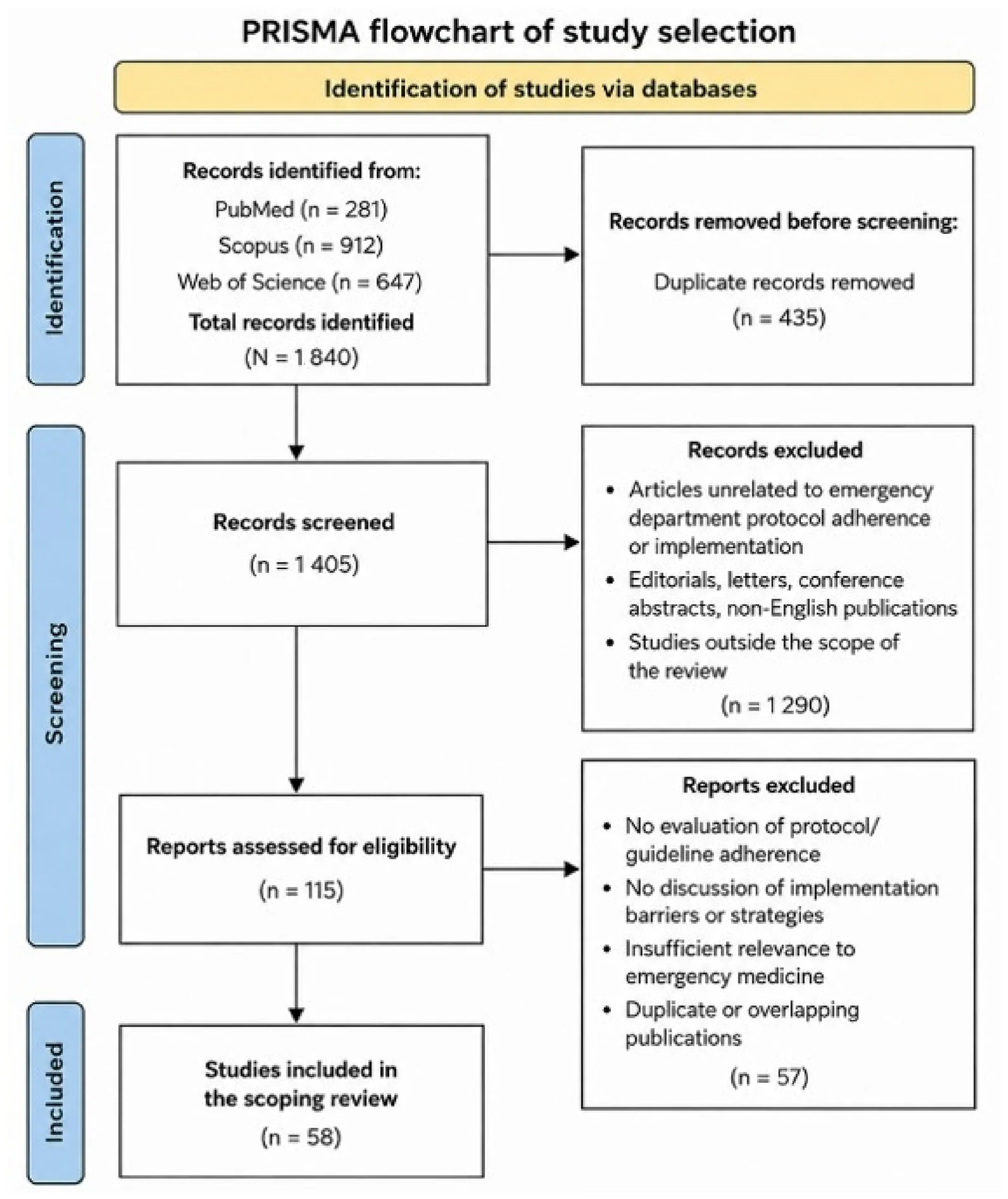
PRISMA-informed flow diagram illustrating the identification, screening, eligibility assessment, and inclusion of studies in the review.

The PRISMA-ScR informed the search methodology. To improve the transparency and reproducibility, the literature search and reporting of study selection procedures were aligned, where applicable, with PRISMA-ScR and the PRISMA-S extension for reporting literature searches. The study selection process is summarized in the PRISMA-ScR-informed flow diagram (Figure 1).

The reference lists of key reviews, guidelines, and highly relevant implementation studies were screened to identify additional sources. Where feasible, supplementary searches of guideline repositories, professional society websites, and implementation reports were considered to broaden the source identification and improve the coverage of practice-oriented evidence, but no new relevant records were found outside of the three databases we already checked. The authors P.M., A.B., and A.C. were responsible for the database supplementary searches.

The eligibility criteria were defined according to the population, concept, context, evidence type, publication period, language, and full-text availability. The population included emergency clinicians, patients, emergency departments, prehospital teams, acute care teams, and related hospital settings. The core concept was adherence or non-adherence to clinical protocols or clinical practice guidelines, including barriers, facilitators, determinants, and implementation strategies. The context included emergency departments, prehospital care, acute care, and closely related hospital settings that handle patient flow and admissions (Triage Area, Observation Unit/Short-Stay Unit).

The eligible evidence sources included systematic reviews, meta-analyses, scoping reviews, observational studies, qualitative and mixed-methods studies, implementation trials, consensus statements, and clinical practice guidelines. Articles were excluded if they were unrelated to emergency or acute care implementation, focused exclusively on non-clinical administrative processes, lacked a clear methodological description, were unavailable in full text, or consisted of opinion pieces without supporting empirical or guideline-based evidence.

The study selection followed a staged screening process. Titles and abstracts were screened against the predefined eligibility criteria, followed by the full-text assessment of potentially eligible publications.

Data charting focused on the bibliographic characteristics, country or region, clinical setting, population, target protocol or guideline, study design, evidence source type, definition of adherence, adherence outcome measures, reported barriers and facilitators, implementation strategies, and reported process or patient outcomes.

The findings were synthesized descriptively and thematically. Because the included evidence was expected to vary substantially regarding the clinical conditions, adherence definitions, implementation interventions, outcome measures, and study designs, no meta-analysis was planned. The evidence was mapped according to the study characteristics, clinical context, determinants of adherence, implementation strategies, and reported outcomes. Barriers and facilitators were grouped into clinician-related, protocol-related, patient-related, organizational, and health-system domains.

No formal risk-of-bias appraisal was applied across all included studies. The purpose of this review was to map the available evidence and identify knowledge gaps rather than to determine the intervention effectiveness or produce pooled effect estimates. This approach strengthens the transparency and reproducibility while acknowledging potential limitations, including database and language restrictions, variability in adherence definitions, heterogeneity across study designs and clinical contexts, and the absence of formal risk-of-bias assessment.

**Figure 2:**
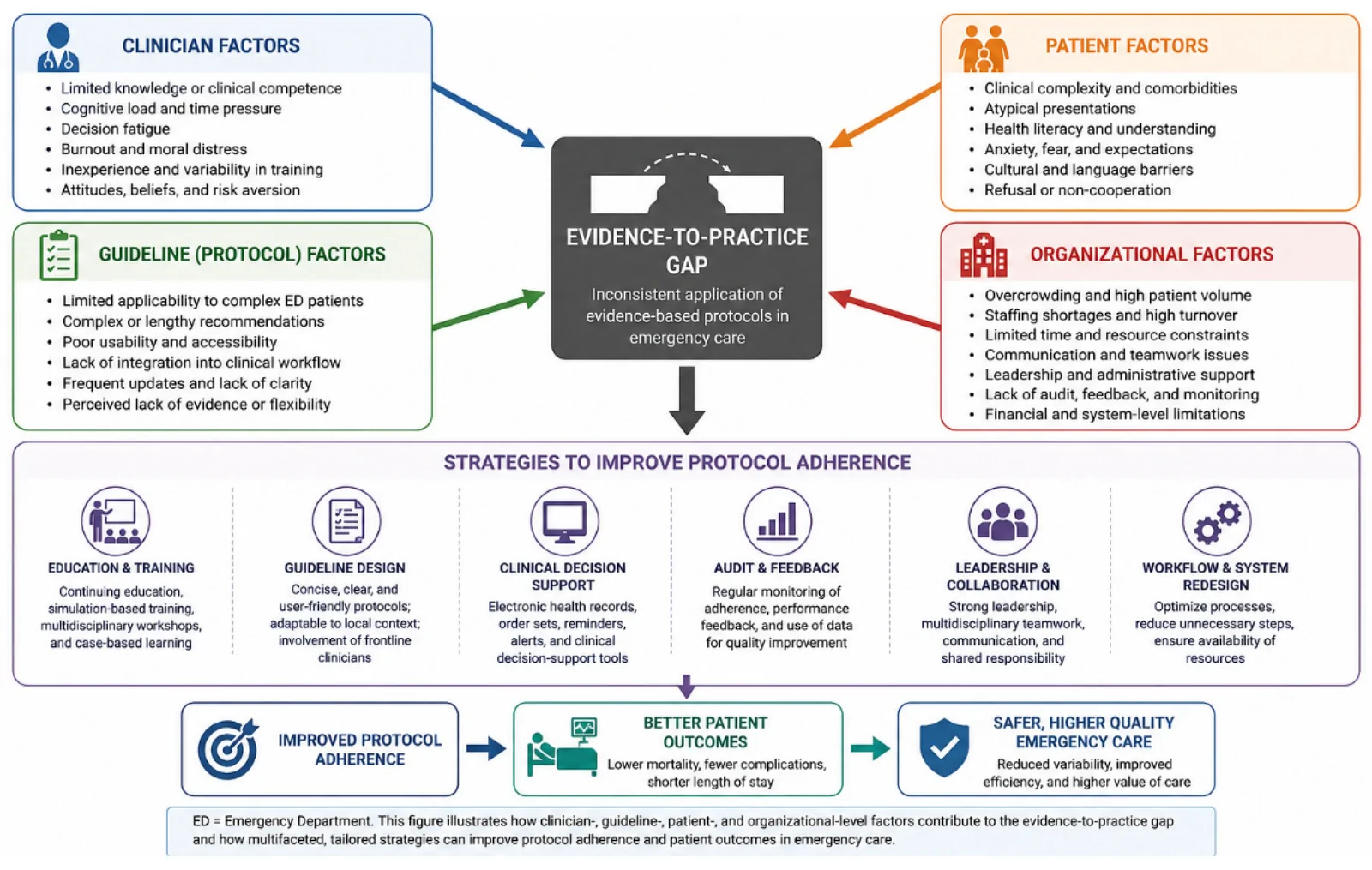
Multilevel determinants of protocol non-adherence in emergency departments and strategies to improve implementation. Source: Conceptualized by the authors and graphically produced using AI. The scientific content, terminology, and final version were reviewed and approved by the authors.

Generative artificial intelligence (GenAI) was used in this paper to assist in the creation of the shapes used in Figure 2.

## 3. Results

### 3.1. Barriers to Protocol Adherence in Emergency Departments

Despite the widespread implementation of evidence-based clinical practice guidelines, adherence to emergency care protocols remains highly variable across healthcare settings. A systematic review by Ebben et al. reported adherence rates ranging from 7.8% to 95% in prehospital emergency care and from 0% to 98% in emergency departments, highlighting a substantial evidence-to-practice gap [16,31]. This variability suggests that patients may be exposed to underuse and overuse of evidence-based interventions, potentially compromising the quality and safety of emergency care. Protocol adherence is influenced by numerous interacting factors, many of which are specific to the emergency department environment and extend beyond individual clinician performance [15,31].

#### 3.1.1. Clinician-Related Factors

Clinician-related factors are consistently recognized as the principal determinants of protocol adherence in emergency medicine. Although evidence-based clinical practice guidelines are designed to standardize patient management and reduce unwarranted variations in care, their effectiveness ultimately depends on the ability of healthcare professionals to integrate these recommendations into routine clinical practice. Contemporary implementation research has consistently demonstrated that clinicians’ knowledge, skills, beliefs, attitudes, motivation, and behavioral characteristics represent the primary determinants of successful guideline implementation, emphasizing that improving protocol adherence requires substantially more than the dissemination of evidence-based recommendations alone [32,33]. Emergency departments represent one of the most challenging environments for protocol implementation. Emergency physicians, nurses, and other members of the multidisciplinary team are required to evaluate undifferentiated patients, rapidly identify life-threatening conditions, and initiate appropriate treatment while simultaneously managing diagnostic uncertainty, fluctuating patient volumes, competing clinical priorities, frequent interruptions, and severe time pressure. These unique characteristics substantially increase the cognitive workload and emotional stress, making the consistent application of standardized clinical protocols particularly difficult in routine practice [10,31,33]. Protocol non-adherence rarely results from a single isolated factor but rather reflects the complex interaction of multiple clinician-related barriers. Recent evidence indicates that these barriers operate at several levels, including individual, guideline-related, and organizational domains, with clinician-related factors consistently identified as the major determinants of successful protocol implementation [10,16,34]. Among the most consistently reported clinician-level barriers are insufficient knowledge of and familiarity with clinical guidelines, limited clinical experience and technical skills, cognitive overload, burnout and fatigue, ineffective communication and teamwork, and individual beliefs and attitudes toward evidence-based practice. Each of these determinants is discussed in the following subsections. Recognizing and addressing these barriers is essential for developing targeted implementation strategies capable of improving protocol adherence, reducing unwarranted practice variation, and ultimately enhancing patient safety and the quality of emergency care [31,33,35,36,37].

#### 3.1.2. Knowledge, Clinical Competence, and Professional Skill

Adequate knowledge of evidence-based clinical practice guidelines is a prerequisite for consistent protocol adherence in emergency medicine. However, awareness of guideline recommendations alone is insufficient to ensure their implementation in routine clinical practice. Behavioral research indicates that knowledge represents only one determinant of guideline adherence, while clinicians’ beliefs, perceived capabilities, and the availability of environmental and organizational resources also influence whether evidence-based recommendations are translated into clinical practice [38]. Multiple studies have identified inadequate familiarity with guideline content, limited understanding of recommended diagnostic and therapeutic pathways, and insufficient technical skills as some of the most common clinician-related barriers to protocol adherence. These deficiencies are particularly relevant in emergency departments, where clinicians must manage diverse time-critical conditions while incorporating frequently updated recommendations into rapid clinical decision-making. In emergency departments, insufficient knowledge or procedural competence may delay the recognition of life-threatening conditions and reduce timely adherence to time-critical protocols such as those for sepsis, acute coronary syndromes, stroke, or major trauma.

Clinical competence extends beyond theoretical knowledge and includes the ability to perform accurate clinical assessment, prioritize diagnostic and therapeutic interventions, formulate appropriate management plans, and communicate effectively with patients and multidisciplinary team members [39,40,41]. In emergency medicine, clinical competence also requires the integration of technical proficiency, clinical reasoning, situational awareness, leadership, and teamwork to ensure safe care in high-acuity environments [42]. In high-acuity emergency settings, insufficient procedural experience or limited confidence in performing recommended interventions may result in deviations from standardized protocols, especially when decisions must be made under severe time pressure and diagnostic uncertainty. Consequently, successful guideline implementation requires sound clinical knowledge and the ability to apply it effectively in complex emergency settings. Maintaining procedural competence has become increasingly challenging, because opportunities to perform high-acuity low-frequency procedures have declined owing to work-hour restrictions, increasing subspecialization, and patient safety initiatives [42]. Continuous professional development represents one of the most consistently reported facilitators of protocol adherence [33,43]. Educational interventions, including continuing medical education, simulation-based training, multidisciplinary workshops, case-based learning, and regular protocol updates, consistently improve clinicians’ knowledge, procedural competence, and confidence in applying evidence-based recommendations. Simulation-based training is particularly valuable in emergency medicine, because it enables healthcare professionals to rehearse the management of rare or high-acuity scenarios while simultaneously strengthening their technical skills, communication, leadership, and team coordination [25,33,44]. However, education alone rarely results in sustained improvements in protocol adherence.

Collectively, these findings suggest that educational interventions should be viewed as one component of a broader behavior-change strategy. Contemporary implementation research indicates that improving guideline adherence requires interventions targeting multiple behavioral determinants, including knowledge, beliefs, self-efficacy, and environmental context, in addition to education [25,32,38].

#### 3.1.3. Cognitive Load, Clinical Decision-Making, and Decision Fatigue

Emergency clinicians routinely make numerous high-stakes decisions under conditions of diagnostic uncertainty, severe time pressure, frequent interruptions, multitasking, and competing clinical priorities. As cognitive demands accumulate throughout a shift, clinicians may experience cognitive overload and decision fatigue, reflecting the progressive depletion of the mental resources required for sustained analytical reasoning. These processes may compromise adherence to evidence-based protocols, particularly during prolonged shifts, periods of overcrowding, or sustained high-intensity clinical activity [45,46,47].

Decision fatigue influences not only the speed but also the quality of clinical reasoning. According to the dual-process theory, physicians alternate between two complementary modes of thinking: an intuitive, rapid, and pattern-recognition process (System 1) and a slower, analytical, and deliberate process (System 2). While System 1 enables the rapid recognition of familiar emergency presentations and the prompt initiation of life-saving interventions, System 2 is essential for complex diagnostic reasoning, differential diagnosis, and the consistent application of evidence-based guidelines. Under conditions of cognitive overload, clinicians increasingly rely on intuitive reasoning because it requires less cognitive effort, whereas analytical reasoning becomes progressively less efficient. Although this adaptive shift facilitates rapid decision-making in familiar situations, excessive reliance on heuristic thinking increases susceptibility to cognitive biases, including premature closure, anchoring, confirmation bias, and availability bias, potentially resulting in diagnostic errors and deviations from guideline-recommended care [46,48,49].

The effects of decision fatigue extend beyond diagnostic reasoning. Cognitive depletion impairs clinicians’ ability to integrate complex clinical information, maintain attention, process information efficiently, and sustain confidence in clinical judgement. Consequently, clinicians may postpone cognitively demanding decisions, request unnecessary confirmatory investigations, adopt more conservative management strategies, or defer responsibility to colleagues or subsequent shifts. Fatigue has also been associated with reduced communication quality, diminished empathy, increased medication and documentation errors, and higher reliance on routine rather than individualized clinical decisions, all of which may indirectly compromise protocol adherence and patient safety [46,50,51]. These findings are supported by a prospective study involving 827 fatigue assessments across 425 emergency department shifts, which demonstrated a significant deterioration in all major dimensions of occupational fatigue during a single shift. Lack of energy showed the highest decline, increasing from a mean score of 3.56 to 5.57 (Cohen’s *d* = 0.64), whereas sleepiness during night shifts increased from 2.58 to 5.30 (Cohen’s *d* = 0.81), highlighting the cumulative impact of prolonged emergency department work and circadian disruption on clinicians’ cognitive performance [52].

Importantly, decision fatigue does not affect all clinical decisions equally. High-acuity emergencies managed using well-established algorithms and multidisciplinary teamwork appear relatively resilient, because standardized protocols reduce the cognitive burden and facilitate rapid decision-making. In contrast, decisions involving diagnostic ambiguity, prolonged deliberation, or lower perceived urgency are more vulnerable to fatigue-related impairment and are therefore more likely to deviate from evidence-based recommendations. Collectively, these findings suggest that optimizing protocol adherence requires not only knowledgeable and clinically competent professionals but also organizational strategies that minimize unnecessary cognitive load, preserve clinicians’ cognitive resources, and support analytical decision-making throughout demanding emergency department shifts [46,52,53].

#### 3.1.4. Burnout, Stress, and Occupational Fatigue

Burnout has become a pervasive occupational syndrome affecting physicians across virtually all medical specialties. In a recent multicenter study, approximately 69% of hospital physicians met the criteria for burnout [54]. These findings suggest that burnout should be regarded as a system-wide challenge rather than a specialty-specific phenomenon. Nevertheless, emergency physicians are exposed to a unique combination of occupational stressors, including shift work, circadian rhythm disruption, emergency department overcrowding, repeated exposure to critically ill patients, frequent interruptions, and sustained cognitive demands [55]. Accordingly, a recent systematic review reported a burnout prevalence ranging from 18% to 71.4% among emergency physicians, whereas depression and occupational stress affected 15.5–19.3% and 19.5–22.7% of clinicians, respectively [56]. Similarly, burnout was identified in 77.8% of emergency medicine trainees in a pilot study evaluating physician well-being and patient experience, highlighting that occupational exhaustion begins early during emergency medicine training [57]. Beyond its impact on clinicians, burnout has been consistently associated with reduced quality of care, impaired patient safety, increased medical errors, lower job satisfaction, and a higher intention to leave emergency medicine [56]. Furthermore, physicians experiencing burnout received significantly lower patient satisfaction scores for communication, listening, courtesy, and concern for patient comfort, demonstrating that clinician well-being directly influences patient-centered care [57].

Fatigue represents the immediate consequence of sustained emergency department work and is increasingly recognized as a precursor to burnout (Patterson et al.). In a prospective study including 827 fatigue assessments performed across 425 emergency department shifts, all major dimensions of occupational fatigue increased significantly over the course of a single shift. Fatigue has therefore been described as a latent hazard in emergency medicine because it progressively impairs attention, communication, multitasking, and clinical decision-making, thereby increasing the susceptibility to protocol deviations and medical errors [52,58]. Psychological distress further compounds these occupational pressures. In a nationwide cross-sectional study involving 15,243 emergency physicians, 35.6% met the criteria for depression. Shift work (OR 1.38, 95% CI 1.20–1.58), poor sleep quality (OR 4.94, 95% CI 4.24–5.75), and workplace violence (OR 3.51, 95% CI 2.94–4.20) were among the strongest independent predictors of depression, underscoring the contribution of organizational and environmental factors to clinicians’ mental health [55]. Furthermore, clinicians with fewer than seven days off per month had almost five-fold higher odds of burnout (adjusted OR 4.70, 95% CI 1.24–17.82), whereas the presence of multiple modifiable risk factors increased the odds of burnout by nearly seven-fold. These findings suggest that work scheduling, recovery time, and workplace safety represent potentially modifiable organizational targets for burnout prevention [59].

Collectively, these findings indicate that burnout, stress, and occupational fatigue should not be regarded solely as clinician well-being issues but as major determinants of protocol adherence, patient safety, and healthcare quality. Consequently, implementation strategies aimed at improving adherence to evidence-based emergency care protocols should incorporate organizational interventions targeting workload, staffing, shift scheduling, recovery time, workplace safety, and psychological support alongside traditional educational initiatives.

### 3.2. Protocol-Related Factors

Although clinician-related determinants play a central role in protocol adherence, successful implementation also depends on the characteristics of the clinical guidelines themselves. Even highly knowledgeable and motivated clinicians may experience difficulties applying recommendations that are complex, insufficiently specific, poorly integrated into emergency department workflows, or inadequately adapted to the heterogeneous patient populations encountered in routine practice. Consequently, guideline-related factors have emerged as an important contributor to protocol non-adherence and should be considered alongside clinician- and organizational-level barriers [31,33].

#### Limited Applicability of Clinical Guidelines to Real-World Emergency Practice

Successful protocol implementation depends not only on the strength of the supporting evidence but also on the usability of clinical recommendations within the emergency department environment. Emergency physicians work under continuous time pressure, frequent interruptions, competing priorities, and rapidly changing clinical conditions. Consequently, protocols that are lengthy, highly prescriptive, require multiple sequential actions, or are poorly integrated into existing workflows are substantially less likely to be implemented consistently than concise, pragmatic, and workflow-oriented recommendations [33]. Guideline implementation should therefore be regarded as an operational challenge as much as an evidence-based practice challenge.

The practical consequences of poor protocol usability are evident across multiple emergency conditions. In hypertensive emergencies, a real-world study demonstrated overall adherence to ACC/AHA recommendations of less than 1%, despite the widespread availability of contemporary guidelines [60]. Complete adherence requires rapid diagnosis, prompt initiation of intravenous antihypertensive therapy, achievement of predefined blood pressure targets within narrow therapeutic windows, continuous hemodynamic monitoring, and avoidance of treatment-induced hypotension. Importantly, hypotensive episodes independently predicted new end-organ injury, indicating that emergency physicians frequently modify guideline recommendations to balance protocol adherence against patient safety. These findings illustrate that protocols requiring intensive monitoring, repeated reassessment, and strict physiological targets may be difficult to implement during routine emergency department care, particularly in overcrowded and resource-limited settings [45,60].

Comparable implementation challenges have been reported for sepsis management. Although approximately three-quarters of European emergency departments reported having institutional sepsis protocols, only 55% consistently completed all elements of the recommended 1-h bundle [61]. Emergency clinicians identified overcrowding, insufficient staffing, delayed recognition of sepsis, limited monitoring capacity, and difficulties integrating multiple time-dependent interventions into the routine workflow as the principal barriers to implementation. Similarly, Reich et al. reported that inconsistent sepsis definitions, uncertainty regarding patient selection, inadequate audit systems, and concerns that rigid bundle implementation could promote unnecessary broad-spectrum antibiotic use further reduced protocol adherence [62]. Collectively, these findings indicate that successful implementation depends not only on protocol availability but also on recommendations that are operationally feasible within the realities of emergency care [61,62].

Evidence from acute stroke further supports this concept. In the Australian T3 implementation trial, a comprehensive implementation strategy incorporating multidisciplinary education, clinical champions, reminders, audit and feedback, and continuous implementation support failed to improve clinician behavior or patient outcomes [63]. The process evaluation demonstrated that the protocol uptake remained limited because the recommendations were perceived as poorly aligned with emergency department workflow, clinicians questioned the supporting evidence for several interventions, physician engagement remained suboptimal, and professional responsibilities between emergency physicians and stroke teams were insufficiently defined. These findings emphasize that even comprehensive implementation programs are unlikely to improve adherence unless the recommendations are perceived as practical, clinically relevant, and compatible with routine emergency department practice [63,64].

Taken together, evidence across hypertensive emergencies, sepsis, and acute stroke demonstrates that protocol adherence is determined not only by clinicians’ knowledge but also by the implementability of the recommendations themselves. Protocols that are concise; clearly structured; adaptable to patient complexity; integrated into routine clinical workflows; and supported by standardized order sets, electronic decision-support systems, and continuous audit-feedback mechanisms are substantially more likely to achieve sustained implementation than recommendations that are overly complex, cognitively demanding, or operationally difficult to deliver in time-critical emergency settings [33].

### 3.3. Patient-Related Factors

Patient-related factors have been investigated less frequently than clinician- or system-level determinants, but they may influence the timely and consistent implementation of emergency care protocols. Where evidence was derived from settings other than emergency departments, it was considered indirect and hypothesis-generating rather than evidence of an established determinant specific to emergency care. Limited health literacy, language barriers, cultural beliefs, unrealistic expectations, refusal of recommended investigations or treatments, psychological comorbidities, and socioeconomic vulnerability may all interfere with evidence-based decision-making, particularly when rapid patient cooperation is required [33,36,37,65]. Low health literacy represents one of the most consistently reported patient-related barriers. Patients with limited understanding of their condition or the rationale for recommended investigations are less likely to participate effectively in shared decision-making and may delay or refuse time-critical interventions [33,66,67]. Similarly, the discordance between patient expectations and evidence-based management may influence clinicians’ decisions, particularly when patients request investigations or treatments that are not guideline-supported or decline recommended therapies because of personal beliefs or misconceptions regarding the disease severity [33,68].

Psychological factors may further complicate protocol implementation. Fear, anxiety, emotional distress, and uncertainty are common during emergency care and may impair communication, informed consent, and cooperation with diagnostic or therapeutic procedures [61]. Language discordance and multimorbidity represent clinically relevant patient-level challenges in emergency care. A 2024 systematic review and meta-analysis found that adults with a non-dominant language preference had higher odds of emergency department revisits, and verified access to professional interpretation services may help mitigate disparities in subsequent healthcare use [69]. In a population-based cohort comprising 451,291 patients and 1,273,937 emergency department attendances, multimorbidity was associated with higher 30-day mortality, hospital admission, and seven-day emergency department reattendance, as well as longer emergency department stays [47]. These findings support the use of professional interpretation, individualized communication, and tailored care pathways for patients with complex needs.

Socioeconomic circumstances may also influence adherence to emergency protocols. Frailty, multimorbidity, cognitive impairment, limited social support, financial constraints, and competing health priorities may reduce patients’ ability to participate in recommended management pathways or comply with follow-up plans [33,65]. Collectively, these findings suggest that optimizing protocol adherence requires not only evidence-based clinical recommendations but also effective communication, individualized patient education, culturally sensitive care, and the recognition of patients’ psychological and social needs.

### 3.4. Organizational and System-Level Factors

Protocol adherence depends not only on clinicians and guideline characteristics but also on the organizational environment in which emergency care is delivered. Time constraints, emergency department overcrowding, heavy workload, inadequate staffing, insufficient funding, and the limited availability of diagnostic equipment, medications, specialist support, and monitoring resources consistently reduce clinicians’ ability to implement evidence-based recommendations, particularly during time-critical emergencies [32,33,70,71].

Successful implementation requires adequate organizational infrastructure [71,72]. Poor communication between healthcare professionals, fragmented referral pathways, insufficient leadership engagement, lack of implementation strategies, audit and feedback, and limited integration of guidelines into electronic health records or routine clinical workflows have all been associated with lower protocol adherence. Conversely, standardized care pathways, electronic clinical decision-support systems, multidisciplinary collaboration, adequate technical support, and strong institutional leadership facilitate sustained implementation [33,44,65,73].

Broader health-system factors may further compromise implementation. Language barriers, cultural diversity, fragmented healthcare networks, unequal distribution of healthcare resources, and conflicting healthcare policies may hinder communication and continuity of evidence-based care [33,43,73,74]. Collectively, these findings indicate that improving protocol adherence requires organizational investment, adequate resources, and supportive health policies alongside clinician education and high-quality clinical guidelines. The determinants of protocol non-adherence operate at multiple levels and collectively contribute to the persistent evidence-to-practice gap in emergency care. The principal barriers and evidence-based implementation strategies identified in the literature are summarized in Table 1.

## 4. Discussion

### 4.1. Strategies to Improve Protocol Adherence

Given the multifactorial nature of protocol non-adherence, implementation programs frequently combine educational, organizational, behavioral, and technological components. Across the included literature, multifaceted approaches were commonly reported; however, the heterogeneity in settings, intervention components, adherence measures, and outcomes prevents a firm conclusion that they are superior to isolated interventions [31,32].

Multifaceted interventions were not uniformly successful. The Australian T3 acute stroke implementation trial combined multidisciplinary education, clinical champions, reminders, audit and feedback, and ongoing implementation support, but it did not improve clinician behavior or patient outcomes [63]. Its process evaluation identified limited workflow compatibility, uncertainty regarding the supporting evidence, insufficient physician engagement, and unclear professional responsibilities as important barriers to uptake [63,64]. These findings indicate that combining multiple intervention components does not itself ensure successful implementation; contextual fit, stakeholder engagement, role clarity, and operational feasibility may be equally important.

Educational interventions remain a fundamental component of implementation. Continuing medical education, simulation-based training, multidisciplinary workshops, educational outreach, and regular protocol updates have been reported to improve clinicians’ knowledge, procedural competence, and familiarity with evidence-based recommendations. However, the passive dissemination of guidelines or educational materials has a limited impact on clinician behavior. Educational initiatives are considerably more effective when integrated with audit and feedback, local opinion leaders, and continuous performance evaluation [31,32,75,77,79].

Implementation strategies should also focus on improving the usability of clinical guidelines within routine emergency practice. Recommendations that are concise, clearly structured, adaptable to local practice, and compatible with existing workflows are more likely to be adopted than lengthy or highly prescriptive protocols. Involving frontline clinicians in guideline development and local adaptation further increases acceptance, ownership, and long-term adherence [32,33,76,80].

Technological and organizational support have been reported to play an equally important role. Electronic health records, computerized clinical decision-support systems, standardized order sets, automated reminders, and structured documentation can reduce cognitive workload and facilitate the real-time application of guideline recommendations. At the organizational level, adequate staffing, sufficient financial and technical resources, multidisciplinary collaboration, clear leadership, standardized care pathways, and regular audit-feedback programs can create an environment that supports sustainable implementation of evidence-based practice [32,33,80,81,82].

Importantly, implementation strategies should be tailored to the specific barriers identified within each emergency department rather than universally applied. A local assessment of organizational constraints, clinician needs, workflow characteristics, and available resources allows implementation programs to target the determinants most responsible for non-adherence. Current implementation science therefore supports a context-specific multifaceted approach in which education, organizational redesign, technological support, and continuous quality improvement operate synergistically to narrow the evidence-to-practice gap in emergency medicine [31,32,80]. The interaction between these barriers and the corresponding implementation strategies is illustrated conceptually in Figure 2.

Recent reviews have examined selected components of clinical guideline implementation relevant to emergency care. Alrashdi et al. identified individual-, guideline-, and organizational-level determinants of guideline implementation among nurses working in emergency departments and critical care units, including limited knowledge and training, guideline complexity, resource constraints, and insufficient leadership support [10]. Similarly, Zhou et al. emphasized the influence of resources, education, audit and feedback, and organizational leadership, although their umbrella review addressed healthcare settings broadly rather than emergency departments specifically [83]. Other emergency medicine reviews have focused on narrower aspects, including the deimplementation of low-value care [84], escalation-of-care processes [85], and the limited inclusion of patients with limited English proficiency in acute stroke research [78]. The principal contribution of the present scoping review is that it brings these previously fragmented perspectives together within a single emergency department-specific framework. Specifically, it integrates clinician-related factors, patient-related factors, characteristics of the guidelines and protocols themselves, and organizational and institutional determinants, linking each of these domains to corresponding reported implementation strategies. Thus, rather than examining a single professional group, clinical condition, or implementation problem, the present review provides a broader multilevel synthesis of the factors influencing protocol adherence across emergency care.

We conducted this scoping review in accordance with the PRISMA Extension for Scoping Reviews (PRISMA-ScR) guidelines, and the PRISMA-ScR Checklist can be found in Supplementary Materials.

### 4.2. Limitations

This review has several limitations that should be considered when interpreting its findings. First, although the search strategy was structured and PRISMA-informed, the article was designed as a scoping review rather than a formal systematic review. Accordingly, no protocol was prospectively registered, duplicate independent screening was not specified, and no formal risk-of-bias assessment was applied across the included studies.

Second, the included literature was heterogeneous with respect to the clinical setting, target protocol, adherence definition, study design, implementation strategy, and measured outcomes. This heterogeneity precluded quantitative synthesis and limited the ability to compare effect sizes across studies. Consequently, the conclusions should be interpreted as a thematic synthesis of the available evidence rather than as pooled estimates of intervention effectiveness.

Third, some evidence was drawn from adjacent acute care, prehospital, primary care, or hospital implementation literature when emergency department-specific evidence was limited. Although these sources provide transferable insights into guideline implementation, their applicability to the emergency department environment may vary according to local resources, staffing models, patient populations, and organizational constraints.

Finally, the review may be affected by publication bias, language bias, and selective reporting within the available literature. Studies reporting successful implementation strategies may be more likely to be published than studies showing a limited or no effect. Future systematic reviews with prospective registration, duplicate screening, formal quality appraisal, and transparent flow reporting would help strengthen the evidence base for protocol adherence interventions in emergency care.

Because no formal design-specific critical appraisal was performed, publications of differing methodological robustness contributed to the thematic mapping without quality-based weighting. Consequently, the frequency with which a barrier or strategy was reported should not be interpreted as representing the strength or certainty of evidence. This limitation also prevents reliable conclusions regarding the comparative effectiveness of implementation strategies. In addition, the inclusion of evidence from adjacent clinical settings may have introduced indirectness, while language and publication restrictions may have resulted in the selective omission of relevant findings.

### 4.3. Future Directions

Future research should move beyond documenting the existence of protocol non-adherence and focus on identifying which combinations of implementation strategies are most effective, sustainable, and transferable across different emergency department settings.

The findings of this review indicate that non-adherence results from interacting clinician-, guideline-, patient-, and organizational-level determinants; therefore, future studies should use implementation science frameworks to design, test, and evaluate multifaceted interventions tailored to local barriers.

In particular, pragmatic multicenter trials and hybrid effectiveness–implementation studies are needed to determine not only whether interventions improve adherence but also why they succeed or fail under real-world emergency care conditions.

A priority for future work is the development of clinically usable and workflow-integrated protocols.

Guidelines should be co-designed with frontline emergency physicians, nurses, prehospital clinicians, informatics specialists, and patient representatives to ensure that the recommendations are concise, operationally feasible, adaptable to patient complexity, and compatible with emergency department workflows.

Artificial intelligence-based decision support should currently be regarded as a research priority rather than an established method for improving protocol adherence in emergency departments. Although such tools may support triage, risk stratification, and time-critical decision-making, direct evidence that they improve protocol adherence or patient outcomes in routine emergency practice remains limited. The potential benefits must be balanced against alert fatigue, automation bias, limited model interpretability, poor workflow integration, additional documentation burden, inequitable performance across patient groups, and data-governance concerns. Future studies should prioritize prospective external validation, human-factor assessment, and the evaluation of effects on clinician behavior, protocol adherence, and patient-centered outcomes [86,87].

Future implementation initiatives should incorporate continuous audit and feedback, locally relevant performance indicators, and patient-centered outcomes.

Attention should be paid to overcrowded, resource-limited, and high-volume emergency departments, where organizational constraints may substantially modify the effectiveness of implementation strategies.

These actions may help emergency care systems deliver safer, more reliable, and more equitable care soon.

## 5. Conclusions

Protocol non-adherence in emergency departments remains a major challenge that cannot be attributed solely to individual clinician performance. The evidence reviewed demonstrates that adherence is influenced by the complex interaction of clinician-related, guideline-related, patient-related, and organizational factors. Consequently, even well-designed evidence-based recommendations may be difficult to implement consistently within the dynamic, time-critical, and resource-constrained environment of emergency care.

Improving protocol adherence therefore requires more than guideline dissemination or clinician education alone. Its successful implementation depends on practical and adaptable recommendations, continuous professional training, effective multidisciplinary collaboration, organizational support, audit and feedback, and the integration of clinical decision-support tools into routine emergency department practice. Implementation strategies should be tailored to locally identified barriers and embedded within existing clinical processes to achieve sustainable improvements. Future efforts should focus not only on developing new clinical guidelines but also on optimizing their implementation in routine emergency care. Advances in digital health, including electronic clinical decision-support systems and artificial intelligence, may further facilitate evidence-based decision-making and reduce the persistent evidence-to-practice gap. Ultimately, improving protocol adherence will require evidence-based recommendations that are scientifically robust, clinically applicable, and seamlessly integrated into the realities of emergency department practice.

## Figures and Tables

**Table 1:** Key barriers to protocol adherence and implementation strategies in emergency departments.

Domain	Main Barriers	Evidence-Based Implementation Strategies	Relevant Included Studies
Clinician factors	Knowledge deficits, limited experience, cognitive overload, decision fatigue, burnout, communication problems	Continuing education, simulation training, multidisciplinary learning, clinical champions	[21,38,39,40,41,42,46,48,49,51,53,54,55,56,57,58,59,70,73]
Guideline factors	Complex recommendations, poor usability, limited applicability, poor workflow integration	Simplified protocols, local adaptation, user-centered guideline design, integration into EHR	[16,17,19,34,43,60,61,62,63,64,71,72,75]
Patient factors	Low health literacy, anxiety, cultural/language barriers, refusal, multimorbidity	Shared decision-making, patient education, interpreters, individualized communication	[47,65,66,67,69,74,76,77,78]
Organizational factors	Overcrowding, staffing shortages, lack of resources, poor leadership, absent audit	Audit and feedback, leadership engagement, decision-support tools, workflow redesign, adequate staffing	[8,10,15,23,24,25,31,32,33,35,36,37,44,71,79,80]

## Data Availability

The data presented in this study are available on request from the corresponding author.

## Supplementary Materials

The following supporting information can be downloaded at: https://www.mdpi.com/article/10.3390/jcm15166420/s1, Supplementary Table S1. Characteristics of the 58 Publications Included in the Scoping Review, Supplementary Table S2. PRISMA-ScR Checklist, Search query and records from PubMed_Timeline_Results_by_Year, complete Record list pubmed-emergencyd-set search.
