# Supplementary material for: Barriers to Protocol Adherence in Emergency Departments and Evidence-Based Strategies for Successful Implementation: A Scoping Review
Source: J Clin Med. 2026 Aug 19;15(16):6420. doi: 10.3390/jcm15166420 (PMC13513292; doi:10.3390/jcm15166420)
Supplement: Supplementary file 1 [file jcm-15-06420-s001.zip › PUBMED Search strategy.pdf]

("emergency department") AND ("clinical protocol" AND "clinical practice guideline" AND "protocol adherence") AND (implementation OR compliance) OR (barriers OR facilitators) AND (overcrowding AND burnout AND workflow OR communication) AND ("patient safety" OR "quality of care")

Filters applied: Full text, Review, Scoping Review, Systematic Review, English, Humans, Exclude preprints, from 2020/1/1 - 2026/6/1.

MY CUSTOM FILTERS
Edit custom filters

RESULTS BY YEAR
Reset

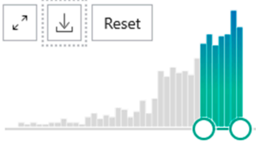

PUBLICATION DATE
- 1 year
- 5 years
- 10 years
- From 2020/1/1 to 2026/6/1 (selected)

TEXT AVAILABILITY
- [ ] Abstract
- [ ] Free full text
- [x] Full text

ARTICLE ATTRIBUTE
- [ ] Associated data

ARTICLE TYPE
- [ ] Books and Documents
- [ ] Clinical Trial
- [ ] Clinical Trial Protocol
- [ ] Collected Work
- [ ] Consensus Statement
- [ ] Controlled Clinical Trial

281 results

Filters applied: Full text, Review, Scoping Review, Systematic Review, English, Humans, Exclude preprints, from 2020/1/1 - 2026/6/1. Clear all

1 Cite
Transport of the Open Abdomen: A Review.
Heffernan M, Taylor B, Cowan S, Newberry R, Lauria M.
Air Med J. 2026 Jul-Aug;45(4):381-384. doi: 10.1016/j.amj.2026.02.012. Epub 2026 Mar 28.
PMID: 42331493 Review.
OBJECTIVE: An open abdomen (OA) is a critical surgical condition in which the abdominal wall is intentionally left open to prevent or manage intra-abdominal hypertension or abdominal compartment syndrome or **facilitate** staged re-exploration. Although OA management is well d ...

2 Cite
Beyond Broca and Wernicke: Epilepsy surgery in the language areas.
Barba C, De Benedictis A, Pelliccia V, Tortora D, Melani F, Consales A, Battaglia DI, Cesaroni E, Ferri L, Noris A, Pugnaghi M, Rizzi M, Bertini G, Villani F, Tassi L; Commission of Epilepsy Surgery, Italian League against Epilepsy.
Epileptic Disord. 2026 Jun;28(3):581-601. doi: 10.1002/epd2.70282. Epub 2026 May 30.
PMID: 42217160 Free PMC article. Review.
The use of all available cortical stimulation methods, combined with test batteries targeting different language subdomains and designed to avoid the induction of post-discharges, appears to be the optimal approach for maximizing **patient safety**. The preservation of ...

3 Cite
Human Factors and Deterioration: What Success Factors Facilitate Escalation of Care and How Can These Be Applied More Reliably?
Rutherfoord C, Jassal C, Galloway R.
Br J Hosp Med (Lond). 2026 May 18;87(5):53978. doi: 10.31083/BJHM53978.
PMID: 42216613 Free article. Review.
Timely escalation of care is critical in preventing adverse **patient** outcomes, yet **barriers** such as poor **communication**, role ambiguity, and hierarchical challenges persist. This article explores key human factors influencing escalation, including structured ...

4 Cite
Language barriers, mobility, and health equity: clinical risks, economic burden, and policy options for universal language access.
Pena-Díaz C.
Int J Equity Health. 2026 May 28;25(1):178. doi: 10.1186/s12939-026-02895-y.
PMID: 42210359 Free PMC article. Review.
