# Supplementary material for: Barriers to Protocol Adherence in Emergency Departments and Evidence-Based Strategies for Successful Implementation: A Scoping Review
Source: J Clin Med. 2026 Aug 19;15(16):6420. doi: 10.3390/jcm15166420 (PMC13513292; doi:10.3390/jcm15166420)
Supplement: Supplementary file 1 [file jcm-15-06420-s001.zip › Supplementary_Table_S1_58_Included_Publications.pdf]

Supplementary Table S1. Characteristics of the 58 Publications Included in the Scoping Review.

The table lists the 58 publications mapped in the review, including bibliographic identification and study/evidence-source type. Citation numbers correspond to the manuscript reference list.

Citation	Author(s)	Year	Study title	Journal/source	Study design / evidence type
[8]	Sartini, M. et al.	2022	Overcrowding in Emergency Department: Causes, Consequences, and Solutions—A Narrative Review	Healthcare (Basel)	Narrative review
[10]	Alrashdi, M.H.; Quinn, B.; Clarke, S.A	2026	Barriers and Facilitators in Implementing Clinical Practice Guidelines Among Nurses in Emergency Departments and Critical Care Units: A Systematic Review	J Clin Nurs	Systematic review
[15]	Gawthorne, J. et al.	2025	Barriers and Enablers to Nurse-Initiated Care in Emergency Departments: An Embedded Mixed Methods Survey Study	Australasian Emergency Care	Embedded mixed-methods survey study
[16]	Ebben, R.H. et al.	2013	Adherence to Guidelines and Protocols in the Prehospital and Emergency Care Setting: A Systematic Review	Scand J Trauma Resusc Emerg Med	Systematic review
[17]	Rhodes, A. et al.	2015	The Surviving Sepsis Campaign Bundles and Outcome: Results from the International Multicentre Prevalence Study on Sepsis (the IMPreSS Study)	Intensive Care Med	International multicentre prevalence/observational study
[18]	Kim, H.-J. et al.	2024	Sepsis Alert Systems, Mortality, and Adherence in Emergency Departments: A Systematic Review and Meta-Analysis	JAMA Netw Open	Systematic review and meta-analysis
[19]	French, W.J. et al.	2022	Emergency Interhospital Transfer of Patients With ST-Segment–Elevation Myocardial Infarction: Call 9-1-1—The American Heart Association Mission: Lifeline Program	Journal of the American Heart Association	Program-based observational study
[20]	Alghamdi, A.S. et al.	2023	Shortening Door-to-Balloon Time: The Use of Ambulance versus Private Vehicle for Patients with ST-Segment Elevation Acute Myocardial Infarction	OAEM	Comparative observational study
[21]	van Rein, E.A.J. et al.	2020	The Role of Emergency Medical Service Providers in the Decision-Making Process of Prehospital Trauma Triage	Eur J Trauma Emerg Surg	Review of prehospital trauma triage decision-making evidence
[24]	Morley, C. et al.	2018	Emergency Department Crowding: A Systematic Review of Causes, Consequences and Solutions	PLOS ONE	Systematic review
[25]	Pearce, S. et al.	2023	Emergency Department Crowding: An Overview of Reviews Describing Measures Causes, and Harms	Intern Emerg Med	Overview of reviews
[26]	Pereira, V.C. et al.	2022	Strategies for the Implementation of Clinical Practice Guidelines in Public Health: An Overview of Systematic Reviews	Health Res Policy Syst	Overview of systematic reviews

Citation	Author(s)	Year	Study title	Journal/source	Study design / evidence type
[35]	Ebben, R.H.A. et al.	2018	Effectiveness of Implementation Strategies for the Improvement of Guideline and Protocol Adherence in Emergency Care: A Systematic Review	BMJ Open	Systematic review
[36]	Fischer, F. et al.	2016	Barriers and Strategies in Guideline Implementation—A Scoping Review	Healthcare	Scoping review
[37]	Wang, T. et al.	2023	Barriers and Enablers to Implementing Clinical Practice Guidelines in Primary Care: An Overview of Systematic Reviews	BMJ Open	Overview of systematic reviews
[38]	Arts, D.L. et al.	2016	Reasons for Intentional Guideline Non-Adherence: A Systematic Review	International Journal of Medical Informatics	Systematic review
[39]	Alweis, R. et al.	2014	An Initiative to Improve Adherence to Evidence-Based Guidelines in the Treatment of URIs, Sinusitis, and Pharyngitis	J Community Hosp Intern Med Perspect	Quality-improvement / guideline-implementation study
[40]	Egerton, T. et al.	2017	A Systematic Review and Evidence Synthesis of Qualitative Studies to Identify Primary Care Clinicians' Barriers and Enablers to the Management of Osteoarthritis	Osteoarthritis and Cartilage	Systematic review and qualitative evidence synthesis
[41]	Rubio-Valera, M. et al.	2014	Barriers and Facilitators for the Implementation of Primary Prevention and Health Promotion Activities in Primary Care: A Synthesis through Meta-Ethnography	PLoS One	Meta-ethnography / qualitative evidence synthesis
[42]	Stewart, D. et al.	2023	A Scoping Review of Theories Used to Investigate Clinician Adherence to Clinical Practice Guidelines	Int J Clin Pharm	Scoping review
[43]	Ghanbari, A. et al.	2017	Assessing Emergency Nurses' Clinical Competency: An Exploratory Factor Analysis Study	Iran J Nurs Midwifery Res	Exploratory factor-analysis study
[44]	Lindström, V.; Falk, A.-C	2023	Emergency Care Nurses' Self-Reported Clinical Competence before and after Postgraduate Education - a Cross-Sectional Study	International Emergency Nursing	Cross-sectional study
[45]	Tan, K.; Black, B.P	2018	A Systematic Review of Health Care Provider-Perceived Barriers and Facilitators to Routine HIV Testing in Primary Care Settings in the Southeastern United States	J Assoc Nurses AIDS Care	Systematic review
[46]	Katyal, A.; Krishnan S, V	2025	Skill Progression Models in Emergency Medicine Training: A Narrative Review and the Development of the OASIS Framework	Int J Emerg Med	Narrative review / framework development
[47]	Ezeani, N	2016	Guideline Use in Asthma Management in Primary Care Setting: A Systematic Review	Walden Dissertations and Doctoral Studies	Systematic review
[48]	Unverzagt, S. et al.	2014	Strategies for Guideline Implementation in Primary Care Focusing on Patients with Cardiovascular Disease: A	Fam Pract	Systematic review

Citation	Author(s)	Year	Study title	Journal/source	Study design / evidence type
			Systematic Review		
[50]	Ozkaynak, M. et al.	2025	Clinical Decision Making and Use of Clinical Decision Support When Clinicians Are Fatigued in an Emergency Department: A Qualitative Study	Appl Clin Inform	Qualitative study
[51]	Blayney, M.C. et al.	2024	Multimorbidity and Adverse Outcomes Following Emergency Department Attendance: Population Based Cohort Study	bmjmed	Population-based cohort study
[52]	Schiessl, L. et al.	2026	Intensive Care Physicians' Experiences of Decision Fatigue and Characteristics of Vulnerable Clinical Decisions	Sci Rep	Qualitative study
[53]	Tay, S.W.; Ryan, P.; Ryan, C.A	2016	Systems 1 and 2 Thinking Processes and Cognitive Reflection Testing in Medical Students	Can Med Educ J	Cross-sectional educational study
[55]	Batur, A	2025	Analysis of Factors Affecting Fatigue in Emergency Medicine Residents: A Nationwide, Cross-Sectional, Descriptive Study	Turk J Emerg Med	Nationwide cross-sectional descriptive study
[57]	Chen, J. et al.	2014	Fatigue and Recovery in 12-Hour Dayshift Hospital Nurses	Journal of Nursing Management	Observational study
[58]	Ghaseminejad, F. et al.	2024	Differences in Physician Burnout by Specialty: A Cross-Sectional Survey	Perm	Cross-sectional survey
[59]	Chen, Y. et al.	2022	Prevalence and Predictors of Depression among Emergency Physicians: A National Cross-Sectional Study	BMC Psychiatry	National cross-sectional study
[60]	Jachmann, A. et al.	2025	Burnout, Depression, and Stress in Emergency Department Nurses and Physicians and the Impact on Private and Work Life: A Systematic Review	JACEP Open	Systematic review
[61]	Lu, D.W. et al.	2018	Emergency Medicine Trainee Burnout Is Associated With Lower Patients' Satisfaction With Their Emergency Department Care	AEM Educ Train	Observational association study
[62]	Klinefelter, Z. et al.	2023	Shift Happens: Emergency Physician Perspectives on Fatigue and Shift Work	Clocks & Sleep	Qualitative survey study
[63]	Kraus, M.; Fischer, M	2025	Burnout in the Emergency Department: Survey of Prevalence and Modifiable Risk Factors	West J Emerg Med	Cross-sectional survey
[64]	Posen, A. et al.	2022	Poor Guideline Adherence in a Real-World Evaluation of Hypertensive Emergency Management	The American Journal of Emergency Medicine	Real-world observational evaluation
[65]	Bolanaki, M. et al.	2025	Current Sepsis Management Practices in European Emergency Departments: The ISG-Emergency Department European Survey	Eur J Emerg Med	European cross-sectional survey
[66]	Reich, E.N.; Then, K.L.; Rankin, J.A	2018	Barriers to Clinical Practice Guideline Implementation for Septic Patients in the Emergency Department	Journal of Emergency Nursing	Implementation/barriers study
[67]	McInnes, E. et al.	2020	Process Evaluation of an Implementation Trial to Improve	Implement Sci	Qualitative process evaluation of an implementation trial

Citation	Author(s)	Year	Study title	Journal/source	Study design / evidence type
			the Triage, Treatment and Transfer of Stroke Patients in Emergency Departments (T3 Trial): A Qualitative Study		
[68]	Craig, L.E. et al.	2016	Identifying the Barriers and Enablers for a Triage, Treatment, and Transfer Clinical Intervention to Manage Acute Stroke Patients in the Emergency Department: A Systematic Review Using the Theoretical Domains Framework (TDF)	Implement Sci	Systematic review using TDF
[69]	Rushforth, B. et al.	2016	Barriers to Effective Management of Type 2 Diabetes in Primary Care: Qualitative Systematic Review	Br J Gen Pract	Qualitative systematic review
[70]	Williams, R.; Moeller, L.; Willis, S	2018	Barriers and Enablers to Improved Access to Health Information for Patients with Low Health Literacy in the Radiotherapy Department	Radiography	Qualitative study
[71]	Shahid, R. et al.	2022	Impact of Low Health Literacy on Patients' Health Outcomes: A Multicenter Cohort Study	BMC Health Serv Res	Multicenter cohort study
[73]	Chu, J.N. et al.	2024	Association between Language Discordance and Unplanned Hospital Readmissions or Emergency Department Revisits: A Systematic Review and Meta-Analysis	BMJ Qual Saf	Systematic review and meta-analysis
[74]	Boltena, M.T. et al.	2026	Barriers to and Facilitators of Adherence to Evidence-Based Standard Antimicrobial Treatment Guidelines among Physicians in Ethiopia: A Formative Qualitative Study	Sci Rep	Formative qualitative study
[75]	Thoonsen, A.C. et al.	2025	Nationwide Guideline Implementation: A Qualitative Study of Barriers and Facilitators from the Perspective of Guideline Organizations	BMC Health Serv Res	Qualitative study
[76]	Thoonsen, A.C. et al.	2024	Stimulating Implementation of Clinical Practice Guidelines in Hospital Care from a Central Guideline Organization Perspective: A Systematic Review	Health Policy	Systematic review
[77]	Chaica, V.; Marques, R.; Pontífice-Sousa, P	2024	ISBAR: A Handover Nursing Strategy in Emergency Departments, Scoping Review	Healthcare	Scoping review
[78]	Davuluri, K. et al.	2022	Patient Perspectives of the Climate of Diversity, Equity, and Inclusion in the Emergency Department	JACEP Open	Cross-sectional patient-perspective study
[79]	Barosi, G	2006	Strategies for Dissemination and Implementation of Guidelines	Neurol Sci	Narrative/practice review
[80]	Smeets, M. et al.	2016	Improving Care for Heart Failure Patients in Primary Care, GPs' Perceptions: A Qualitative Evidence Synthesis	BMJ Open	Qualitative evidence synthesis
[81]	Briedé, S. et al.	2024	Physicians' Training and Patient	BMJ Open Quality	Quality-improvement /

Citation	Author(s)	Year	Study title	Journal/source	Study design / evidence type
			Education Initiative to Improve Quality of Care Decision Communication at the Emergency Department		educational intervention
[82]	Zeidan, A.J. et al.	2023	Limited English Proficiency as a Barrier to Inclusion in Emergency Medicine-Based Clinical Stroke Research	J Immigrant Minority Health	Retrospective review of clinical stroke research studies
[83]	Joosen, M.C. et al.	2015	Improving Occupational Physicians' Adherence to a Practice Guideline: Feasibility and Impact of a Tailored Implementation Strategy	BMC Med Educ	Feasibility and implementation-impact study
[84]	Mostafa, R.; El-Atawi, K.	2024	Strategies to Measure and Improve Emergency Department Performance: A Review	Cureus	Narrative review
